# Novel, First-in-Human, Oral PCLX-001 Treatment in a Patient with Relapsed Diffuse Large B-Cell Lymphoma

**DOI:** 10.3390/curroncol29030158

**Published:** 2022-03-13

**Authors:** Randeep Sangha, Neal M. Davies, Afshin Namdar, Michael Chu, Jennifer Spratlin, Erwan Beauchamp, Luc G. Berthiaume, John R. Mackey

**Affiliations:** 1 Department of Oncology, Faculty of Medicine and Dentistry, University of Alberta, Edmonton, AB T6G1Z2, Canada; michael.chu@albertahealthservices.ca (M.C.); jennifer.spratlin@albertahealthservices.ca (J.S.); john.mackey@pacylex.com (J.R.M.); 2 Faculty of Pharmacy & Pharmaceutical Sciences, University of Alberta, Edmonton, AB T6G2H1, Canada; ndavies@ualberta.ca; 3 Department of Cell Biology, Faculty of Medicine and Dentistry, University of Alberta, Edmonton, AB T6G2H7, Canada; namdarme@ualberta.ca (A.N.); luc.berthiaume@ualberta.ca (L.G.B.); 4 Pacylex Pharmaceuticals Inc., Edmonton, AB T5J4P6, Canada; erwan@pacylex.com

**Keywords:** N-myristoyltransferase inhibitors, PCLX-001, non-Hodgkin lymphoma, phase I, first-in-human, dose escalation, pharmacokinetics, pharmacodynamic assay

## Abstract

Patients with relapsed or refractory diffuse large B-cell lymphoma (DLBCL) have limited treatment options, particularly if they are transplantation or chimeric antigen receptor (CAR) T-cell ineligible, and novel therapeutics are needed. An 86-year-old woman with relapsed DLBCL received a novel, first-in-class small molecule inhibitor of N-myristoyltransferase (NMT) as the initial patient on a phase I dose escalation trial. Daily oral administration of 20 mg PCLX-001 tablets produced a pharmacokinetic profile suitable for single daily dosing: rapid oral absorption, followed by an apparent elimination half-life of 16 h, without systemic accumulation of drug by day 15. Pharmacodynamic tests showed no clear change in NMT1 and NMT2 levels or selected NMT substrate Lyn and HGAL protein levels in normal circulating blood mononuclear cells, suggesting a higher dose will be required for normal tissue toxicity. The patient did not experience any dose-limiting toxicities but had disease progression after 28 days of study therapy. Dose escalation continues in other patients in this first-in-human study of a new class of anticancer drug. We conclude that PCLX-001 oral monotherapy has suitable pharmacokinetic parameters for dose escalation, and that higher doses are required to achieve pharmacodynamic evidence of on-target activity in normal tissues. The current protocol is appropriately designed to achieve these ends, and the study proceeds without modification.

## 1. Introduction

Myristoylation, the N-terminal modification of proteins with the fatty acid myristate, is critical for membrane targeting of several hundred human proteins [1], including ones critical for intracellular signaling. Because cancer cells often have increased N- myristoyltransferase (NMT) protein expression, NMTs have been proposed as anti-cancer targets [2], but have never been studied in human clinical trials. PCLX-001 is a potent, small molecule inhibitor of both human NMT proteins, NMT1 and NMT2. Preclinical studies have shown that PCLX-001 markedly inhibits hematologic and lymphoma cell lines in tissue culture, and achieves complete remissions in human cancers grown in immunodeficient mice [3] and tumour responses in solid cancers [4]. Although PCLX-001 has multiple potential mechanisms of action, in B-cell lymphoma models, it inhibits early B-cell receptor (BCR) signaling events critical for survival. In addition to abrogating myristoylation of Src family kinases, PCLX-001 also promotes their degradation and that of numerous non-myristoylated BCR effectors, including c-Myc, NFκB, and P-ERK, leading to cancer cell death in vitro and in xenograft models [3]. The molecule has been extensively investigated in non-clinical safety testing [5], and found suitable for formal drug development in humans.

Herein, we report the first ever patient treated with an NMT inhibitor. PCLX-001 was administered to a woman with relapsed DLBCL in the setting of a formal, phase I dose escalation trial approved by Health Canada. Pharmacokinetic and pharmacodynamic endpoints were explored in this first-in-human drug exposure, showing PCLX-001 has pharmacokinetic properties suitable for continued development as an oral, once daily, cancer therapy.

## 2. Materials and Methods

### 2.1. PCLX-001—The Investigational Agent

**Figure 1:**
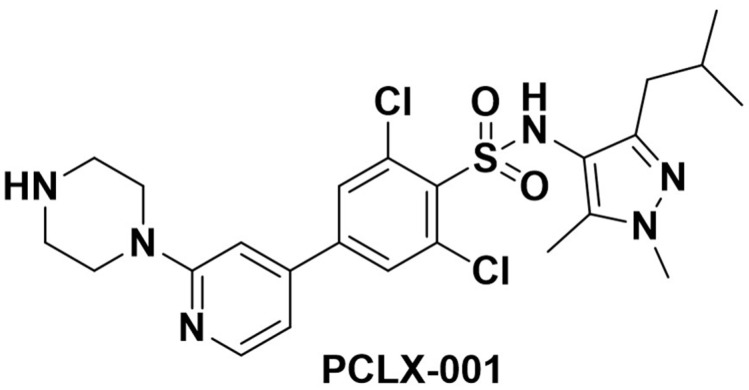
Chemical Structure of PCLX-001 (2,6-dichloro-N-(3-isobutyl-1,5-dimethyl-1H-pyrazol-4-yl)-4-(2- (piperazin-1-yl)pyridin-4-yl)benzenesulfonamide) C_24_H_30_Cl_2_N_6_O_2_S.

PCLX-001 (Figure 1) is a potent, small molecule inhibitor of human NMT1 and NMT2 proteins. In animal models, it has complete oral bioavailability. There is no significant off-target kinase inhibition, as demonstrated by a KINOMEscan (Fremont, CA, USA) [3]. Preclinical testing reveals no significant hERG interaction and animal non-clinical safety testing showed no cardiac conduction issues. In GLP non-clinical safety testing, diarrhea was dose-limiting [5]. Mechanism of action studies show that while NMT inhibition can affect approximately 600 human proteoforms [1], in models of human lymphoma Bruton tyrosine kinase (BTK), modification and downstream signaling was profoundly inhibited in the presence of PCLX-001 [3].

### 2.2. Clinical Trial

The “Phase I Trial of PCLX-001 in B-cell Non-Hodgkin Lymphoma and Advanced Solid Malignancies” is registered at clinicaltrials.gov as NCT04836195. It is a phase I dose-escalation study of oral PCLX-001, conducted in a multicenter, non-randomized, open-label, non-controlled design. The study is comprised of two parts: Part A (single-agent dose escalation) and Part B (single-agent expansion cohorts) [6].

The primary endpoints of the study are to assess the safety and tolerability of PCLX-001 and to determine the maximum tolerated (MTD) and/or recommended Phase II dose (RP2D) using a standard 3 + 3 dose escalation phase I design. Pharmacokinetics (PK) will also be evaluated. Secondary and exploratory objectives will be to determine preliminary anti-tumour activity in dose expansion cohorts and to assess the pharmacodynamic (PD) effects of PCLX-001, respectively.

Patients are to receive oral PCLX-001 on 28-day cycle with the first dose level cohort starting at 20 mg po daily and subsequent dose levels escalated to a maximum of 280 mg po daily (dose level 7). No other cancer therapeutics will be allowed while on trial.

The study was conducted according to the guidelines of the Declaration of Helsinki, and approved by the Institutional Review Board of the Cross Cancer Institute (postal code T6G1Z2 and protocol code HREBA CC-21-0157, approved 24 June 2021). Informed consent was obtained from all subjects involved in the study.

### 2.3. Pharmacokinetic Analysis

The primary pharmacokinetic analysis endpoints were:

(i) To determine the time to maximum plasma level (Tmax) of PCLX-001 measured during Cycle 1: Pre-dose on Days 1, 2, 8(±2), 15 (±2), and 22 (±2); Day 1 and Day 15 will also be measured post-dose at 0.5, 1, 2, 4, and 8 h; Cycle 2 pre-dose on Day 1.

Tmax is the time at which the maximum plasma concentration of PCLX-001 is achieved.

(ii) To determine the maximum plasma level (Cmax) of PCLX-001 measured on Cycle 1: Pre-dose on Days 1, 2, 8(±2), 15 (±2), and 22 (±2); Day 1 and Day 15 will also be measured post-dose at 0.5, 1, 2, 4, and 8 h; Cycle 2 pre-dose on Day 1.

Cmax is the maximum plasma concentration of PCLX-001.

The patient was dosed with oral doses of PCLX-001 20 mg. After dosing, the blood samples (3 mL volume) were serially collected into heparinized tubes from a right forearm butterfly cannula. All blood fluid specimens were frozen and later assayed using a validated UHPLC-MS detection method.

PK Solver 2.0 freeware (https://www.boomer.org/boomer/software/pksolver.zip; accessed 26 November 2021) was used to determine the single dose pharmacokinetic parameters using a two-compartment model. The terminal phase rate constant (λz) was estimated by applying exponential linear regression to the concentrations in the terminal phase, and the apparent terminal half-life (t 1/2) was estimated as 0.693/λz. The area under the concentration (AUC) vs. time curve from the time of injection to the last (Clast) measured concentration (AUC0-tlast) was calculated. The AUC extrapolated to infinity (AUC0–∞) was estimated as the sum of AUC0-tlast and Clast/λz on Days 1 and 15. Oral body clearance (CLoral) was calculated as the ratio of the dose to the AUC0–∞. The area under the first moment concentration vs. time curve (AUMC) was estimated as the sum of the area under the C × t vs. t curve from time zero to Clast, plus the ratios of Clast × t/λz and Clast/λz 2. The mean residence time (MRT) was calculated as the ratio of AUMC to AUC0–∞). The apparent volume of distribution of steady-state (Vdss) was calculated as the product of CL × MRT.

### 2.4. Pharmacodynamic Analysis

Peripheral blood was collected from the patient at each sampling point (Screen, Day 1, and Day 28 of treatment) by venipuncture according to normal phlebotomy practice in 2 × 8 mL BD Vacutainer^®^ CPT™ Tubes. Samples were immediately mixed by gentle inversion 10 times and process for PBMC isolation within 1 h. After centrifugation (1800× *g* for 20 min) at room temperature (18–25 °C) in a swinging bucket rotor (swing-out head), the mononuclear cells and platelets (referred to as PBMC) were carefully collected as the whitish layer just above the polyester gel and under the plasma layer, transferred into 9 mL freezing media (8 mL Fetal Bovine Serum and 1 mL DMSO), and stored at −70 °C or below until flow cytometry analysis.

PBMC were recovered by centrifugation (400× *g* for 5 min), washed in RPMI culture media, resuspended in PBS, and prepared for FACS analysis. PBMC were first labeled with Zombie Aqua™ Fixable Viability kit (1/100, 20 min at 4 °C; BioLegend, San Diego, CA, USA) for exclusion of dead cells, blocked with Human TruStain FcX Fc receptor blocking solution (5/100, 10 min at room temperature, BioLegend), then stained for extracellular markers using antibodies ECD-CD3 (clone UCHT1) and PE-Cy7-CD4 (clone SFCI12T4D11) from Beckman Coulter (Missaugua, ON, Canada) PerCp-Cy5.5-CD8 (SK-1) and BV-785CD19 (clone HIB19) from BioLegend, and PE-CF594-CD14 (clone MΦPG) from BD Bioscience (Mississauga, ON, Canada) for 20 min at 4 °C. The cells were then fixed and permeabilized using the eBioscience Intracellular Fixation and Permeabilization buffer set (Invitrogen, Mississauga, ON, Canada) and stained for intracellular markers AF-488-HGAL (clone 1H1-A7, eBioscience), AF-488-Lyn (Lyn-01, ab1890, Abcam, Cambridge, UK), and homemade monoclonal antibodies AF-700-NMT1(clone 6F8D5) and APC-NMT2 (clone 6C5E8), as previously described [4], in parallel with relevant isotype controls for at least 30 min at 4 °C. Prior to applying in the test, intracellular antibodies were first labeled with different fluorochromes using Lightning-Link conjugation kits (Abcam) and titrated to achieve the best concentration. Finally, samples were acquired on a Fortessa X-20 flow cytometer (BD Bioscences, Faculty of Medicine and Dentistry Flow Cytometry Facility, University of Alberta). Data were analyzed using FlowJo software (TreeStar, Ashland, OR, USA, version 10.8).

### 2.5. Toxicity Assessment

The subject was assessed for toxicity using daily diary entries, clinical assessments at baseline, day 8, day 15, day 22, and day 28. Dose-limiting toxicities (DLTs) were defined as the following cycle 1 CTCAE version 5.0 adverse events: Gr 4 platelets, Gr ≥ 3 platelets with bleeding and/or transfusions, Gr 4 ANC for ≥ 7 days, Gr ≥ 3 febrile neutropenia, and Gr ≥ 3 non-hematological toxicity.

### 2.6. Efficacy Assessment

The subject had baseline cross-sectional and PET imaging. Protocol-defined imaging was CT every 2 cycles and PET every 6 cycles or as clinically indicated. Evaluation of response was by the Lugano classification criteria for the evaluation of non-Hodgkin lymphoma.

## 3. Results

### 3.1. Patient Demographics

The patient was an 86-year-old Caucasian woman originally diagnosed in September 2014 with Stage IVA diffuse large B-cell lymphoma (DLBCL) of non-GCB origin using the Hans algorithm (CD10 negative; BCL6, MUM1 positive). *MYC* and *BCL2/BCL6* translocations over age 70 are not routinely performed in Alberta. This lymphoma was manifesting as mesenteric, left retroperitoneal, common iliac lymphadenopathy, and left proximal femur involvement. Bone marrow was negative for lymphoproliferative involvement. The revised International Prognostic Index (R-IPI) was calculated to be 2 (age, stage). She had completed six cycles of R-CHOP (rituximab, cyclophosphamide, doxorubicin, vincristine, prednisone) + ibrutinib or placebo as part of a clinical trial in March 2015 and had a documented complete response. The patient relapsed in July 2021 with biopsy confirming DLBCL, GCB cell of origin (CD10 and MUM1 negative; BCL6 positive). The apparent discrepancy between cell of origin in the primary and relapse may be an anomaly of the MUM1 IHC but this was not further investigated with gene expression profiling. She had Stage IVA disease at relapse with cervical, hilar, and mediastinal lymphadenopathy as well as a lingular soft tissue mass and T10 vertebral involvement. She was not considered a candidate for autologous stem cell transplant (ASCT) or CAR T-cell directed therapy by virtue of age, comorbidities, and patient choice.

### 3.2. PCLX-001 Administration

PCLX-001 was administered orally each morning as two tablets, each containing PCLX-001 10 mg. PCLX-001 was initiated on 14 September 2021, and discontinued on day 29 due to symptomatic back pain with CT confirming progression of lymphadenopathy and T10 soft tissue mass.

### 3.3. Pharmacokinetics

**Figure 2:**
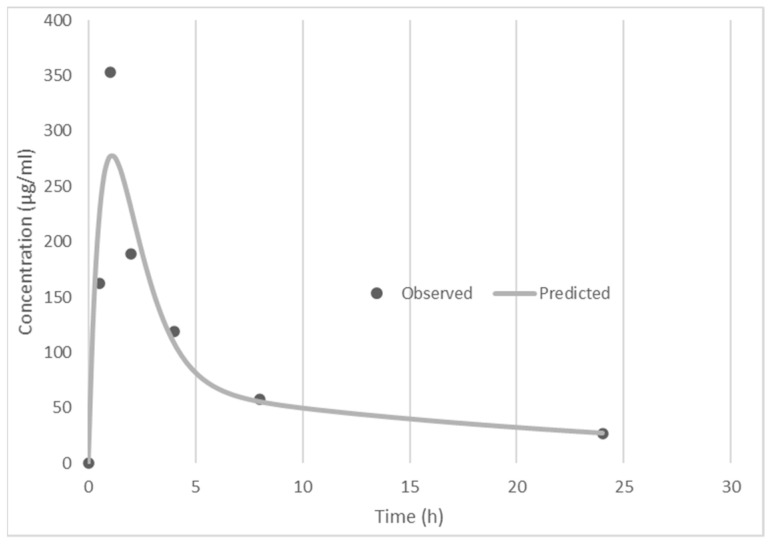
Day 1 Single Dose 20 mg Oral Pharmacokinetic Profile.

Pharmacokinetic analysis was conducted as described above. Figure 2 shows the plasma concentration of PCLX-001 after the initial oral dose. Table 1 shows the pharmacokinetic parameters determined by this analysis. These results show a rapid absorption rate, with peak plasma concentration achieved at 1.1 h after oral administration, a peak plasma concentration of 278 ng/mL, and an apparent terminal half-life of 16 h. Because the first dose AUC and day 15 AUC were very similar (Table 1), there is no evidence of drug accumulation in plasma after repeated daily dosing. These parameters are all supportive of a single daily oral dosing schedule for PCLX-001.

### 3.4. Pharmacodynamics

PCLX-001 target engagement was assessed by flow cytometry. First, we investigated the effect of PCLX-001 on T cell, B cell, and monocyte live populations. As shown in Table 2 and Figure S1, live total T cells (CD3+), CD4+ T cells, CD8+ T cells, B cells (CD19+), and monocytes (CD14+) were only marginally affected by 28 days of PCLX-001 treatment and demonstrated no sign of apparent toxicity in these healthy cell populations (Table 2).

To further investigate the impact of a myristoylation inhibitor in vivo, we measured the protein levels of two myristoylated proteins, Lyn and HGAL. Both protein targets were shown to be significantly decreased in the presence of PCLX-001 in vitro in multiple Burkitt Lymphoma and Diffuse Large B cell lymphoma cell lines [3]. At this dose, no apparent trend was identified for either protein (Table 3 and Figures S2–S5). HGAL expression (Figure 1) was high in CD14+ monocytes but did not decrease after 28 days of treatment (Table 3, Figure S5). The discrepancy in the level of HGAL measured between the Screen sample and Day 1 sample demonstrates that endogenous levels can fluctuate without treatment and this variability does not permit conclusions on the level of HGAL at the end of the treatment. A similar trend was observed for Lyn with high expression in monocytes but no significant decrease in all populations at the end of the first treatment cycle.

Finally, we sought to measure the impact of PCLX-001 treatment on the protein level of the inhibited targets, NMT1 and NMT2, and evaluate if the compound could stabilize the enzyme or induce a cellular response to their inhibition. NMT1 and NMT2 levels were very stable in all cell populations, with the exception of monocytes (Table 3, Figures S2–S5). For each isoform, protein levels increased at the end of the first cycle. This observation must be confirmed in new enrolled patients but could be the result of a physiologic cellular response mechanism to counteract myristoylation inhibition by PCLX-001.

### 3.5. PCLX-001 Toxicities

The patient diary revealed perfect compliance with the medication delivery schedule. The patient experienced no protocol-defined dose-limiting toxicities. Her hematopoietic profile and biochemistry remained normal throughout the trial.

### 3.6. Efficacy

The subject had baseline cross-sectional and PET imaging. Due to increasing patient symptoms of back pain, repeat computed tomography imaging was performed on day 29 and revealed progression of the volume of lymphoma, as defined by the Lugano classification criteria for the evaluation of non-Hodgkin lymphoma. In consequence, the patient discontinued PCLX-001 at that time.

## 4. Discussion

This is the first report of human exposure to a potential new class of anti-cancer drug, inhibitors of N-myristoyltransferase. In this prospective, phase I dose escalation study, the first patient received daily 20 mg oral PCLX-001 for a complete 28-day cycle and experienced no dose-limiting toxicities. This patient had relapsed and pre-treated aggressive non-Hodgkin lymphoma, and although she experienced disease progression despite PCLX-001, there was no evidence of toxicity to hematopoietic function, liver function, or kidney function.

Pharmacokinetic analysis showed rapid absorption of the drug after oral dosing, with peak plasma concentration achieved 1.1 h after oral administration, a peak plasma concentration of 278 ng/mL, and an apparent terminal half-life of 16 h. Because the first dose AUC and day 15 AUC were very similar, there is no evidence of drug accumulation in plasma after repeated dosing. These parameters are all supportive of a single daily oral dosing schedule for PCLX-001. Furthermore, the plasma trough levels, as measured immediately before daily dosing, achieve drug concentrations that approximate the IC50 required to inhibit some PCLX-001-sensitive cultured cancer cell lines.

Pharmacodynamic analyses were conducted on circulating peripheral blood cells, which did not demonstrate clear biological effects on these normal cells. Additional patients and higher drug doses will be required to validate the utility of these markers to guide drug dosing.

This patient potentially had prior exposure to the Bruton tyrosine kinase (BTK) inhibitor ibrutinib (double-blind clinical trial). Some of myristoylation substrates for NMTs, including Lyn and HGAL, are upstream of BTK in the B-cell receptor signaling cascade [3]. Whether prior BTK inhibitors relate to PCLX-001 efficacy will be assessed as additional patients are accrued to this ongoing study.

In aggregate, these findings are strongly supportive of continued evaluation and dose escalation of daily oral PCLX-001 in advanced cancers.

## 5. Conclusions

This is the first report of the therapeutic human use of N-myristoyltransferase inhibitors. PCLX-001, a potent, small molecule inhibitor of human N-myristoyltransferase proteins, was administered in a dose escalation phase I first-in-human study to a woman with relapsed DLBCL. PCLX-001 pharmacokinetics support daily oral dosing, and pharmacodynamic studies suggest a dose higher than 20 mg daily is required to trigger normal tissue toxicities.

## Figures and Tables

**Table 1:** Pharmacokinetic analysis results.

Pharmacokinetic Parameters	Data
Cmax (ng/mL)	278.23
Tmax (h)	1.1
AUC0-tlast (ng/Ml.h)	1701.33
AUC0–∞ (ng/mL.h)\	2355.81
Day 15 AUC0–∞ (ng/mL.h)	2635.63
MRT (h)	18.63
T ½ (h)	16.42
AUMC (ng/mL.h^2^)	43,883.76
CLoral L/h	8.4
Vdss (L)	156.49
Day 22 pre-dose (ng/mL)	51.4

**Table 2:** Impact of PCLX-001 treatment on T cells, B cells, and monocyte population.

Cell Populations	Screening Sample (%)	Day 1 (%)	Day 28 (%)
Total T cells	66.6 ± 0.52	66.7 ± 0.51	70.1 ± 0.50
CD4+ T cells	40.2 ± 0.24	42.5 ± 0.24	46.8 ± 0.22
CD8+ T cells	25.1 ± 0.33	23.1 ± 0.34	22.5 ± 0.31
CD19+ B cells	0.71 ± 0.66	1.18 ± 0.51	0.59 ± 0.50
CD14+ monocytes	5.01 ± 0.4	14.2 ± 0.40	4.51 ± 0.42

PBMC were isolated from patient 1 after 28 days of treatment with PCLX-001 and analysed by flow cytometry. Extracellular staining allowed us to analyse the proportion of live total T cells (CD3+), CD4+ T cells, CD8+ T cells (A), B cells (CD19+), and monocytes (CD14+). Values are expressed as % of population ± CV.

**Table 3:** Impact of PCLX-001 treatment on the intracellular level of HGAL, Lyn, NMT1, and NMT2 in CD4+ T cells, CD8+ T cells, CD19+ B cells, and CD14+ monocytes.

Cell Populations	Screening Sample (%)	Day 1 (%)	Day 28 (%)
**CD4+ T cells**			
HGAL	12.7 ± 0.57	22.1 ± 0.73	8.6 ± 0.58
Lyn	13.7 ± 0.28	20 ± 0.28	28.7 ± 0.37
NMT1	2.99 ± 0.69	4.87 ± 0.63	2.22 ± 0.63
NMT2	22.8 ± 0.33	25.7 ± 0.31	35.3 ± 0.36
**CD8+ T cells**			
HGAL	25 ± 0.61	30.3 ± 0.58	17 ± 0.63
Lyn	17.6 ± 0.17	25.8 ± 0.28	33.3 ± 0.31
NMT1	0.94 ± 0.55	0.85 ± 0.64	0.77 ± 0.38
NMT2	11 ± 0.11	12.7 ± 0.27	18.7 ± 0.33
**CD19+ B cells**			
HGAL	2.44 ± 0.41	11.2 ± 0.51	8.33 ± 0.39
Lyn	8.33 ± 0.29	12.4 ± 0.27	12.1 ± 0.15
NMT1	0.98 ± 0.41	2.65 ± 0.71	2.42 ± 0.42
NMT2	31.8 ± 0.29	29 ± 0.3	33.3 ± 0.24
**CD14+ Monocytes**			
HGAL	83.9 ± 0.64	87.5 ± 0.65	88.3 ± 0.64
Lyn	51.4 ± 0.50	40.5 ± 0.39	66.6 ± 0.59
NMT1	1.59 ± 0.19	1.37 ± 0.33	4.55 ± 0.30
NMT2	6.21 ± 0.45	6.7 ± 1.13	11.7 ± 0.17

PBMC were isolated from patient 1 after 28 days of treatment with PCLX-001 and analysed by flow cytometry. Intracellular staining allowed us to analyse HGAL, NMT1 (A), Lyn, and NMT2 levels in CD4+ T cells, CD8+ T cells (A), B cells (CD19+), and monocytes (CD14+) population. Values are expressed as % of population ± CV.

## Data Availability

Data supporting these results is on file with Pacylex Pharmaceuticals, Inc.

## Supplementary Materials

The following are available online at https://www.mdpi.com/article/10.3390/curroncol29030158/s1, Figure S1: Impact of PCLX-001 treatment on T cells, B cells, and monocyte populations. Figure S2: Impact of PCLX-001 treatment on intracellular levels of NMT1, NMT2, HGAL, and Lyn in the CD4+ T cell population. Figure S3: Impact of PCLX-001 treatment on intracellular levels of NMT1, NMT2, HGAL, and Lyn in CD8+ T cells. Figure S4: Impact of PCLX-001 treatment on intracellular levels of NMT1, NMT2, HGAL, and Lyn in CD19+ B cells. Figure S5: Impact of PCLX-001 treatment on intracellular levels of NMT1, NMT2, HGAL, and Lyn in CD14+ monocytes.
