# Supplementary material for: Novel, First-in-Human, Oral PCLX-001 Treatment in a Patient with Relapsed Diffuse Large B-Cell Lymphoma
Source: Curr Oncol. 2022 Mar 13;29(3):1939–46. doi: 10.3390/curroncol29030158 (PMC8947478; doi:10.3390/curroncol29030158)
Supplement: Supplementary file 1 [file curroncol-29-00158-s001.zip › curroncol-1582452-supplementary.pdf]

Supplementary material

Novel, First-In-Human, Oral PCLX-001 Treatment in a Patient with Relapsed Diffuse Large B-Cell Lymphoma

Randeep Sangha, Neal M. Davies, Afshin Namdar, Michael Chu, Jennifer Spratlin, Erwan Beauchamp, Luc G. Berthiaume and John R. Mackey

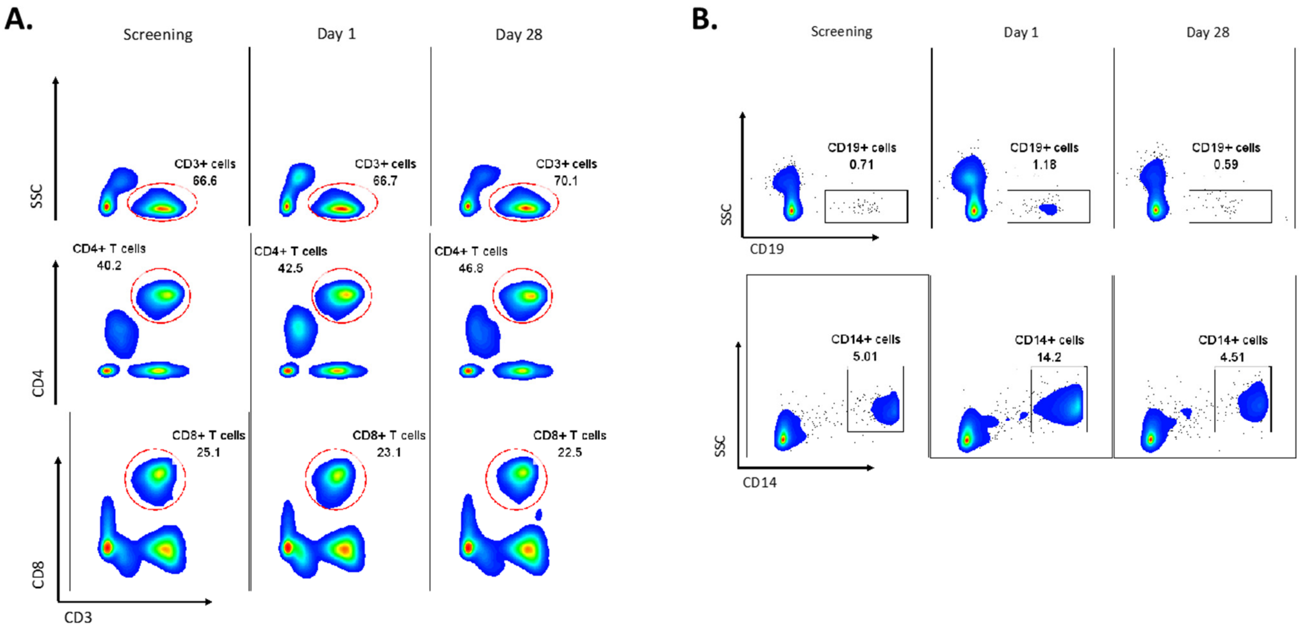

Figure S1. Impact of PCLX-001 treatment on T cell, B cell and monocyte populations.PBMC were isolated from patient 1 during screening, Day 1 and after 28 days of treatment with PCLX-001 and analysed by flow cytometry. Extracellular staining allowed us to analysed proportion of live total T cells (CD3+), CD4+ T cells, CD8+ T cells (**A**), B cells (CD19+) and monocytes (CD14+) (**B**).

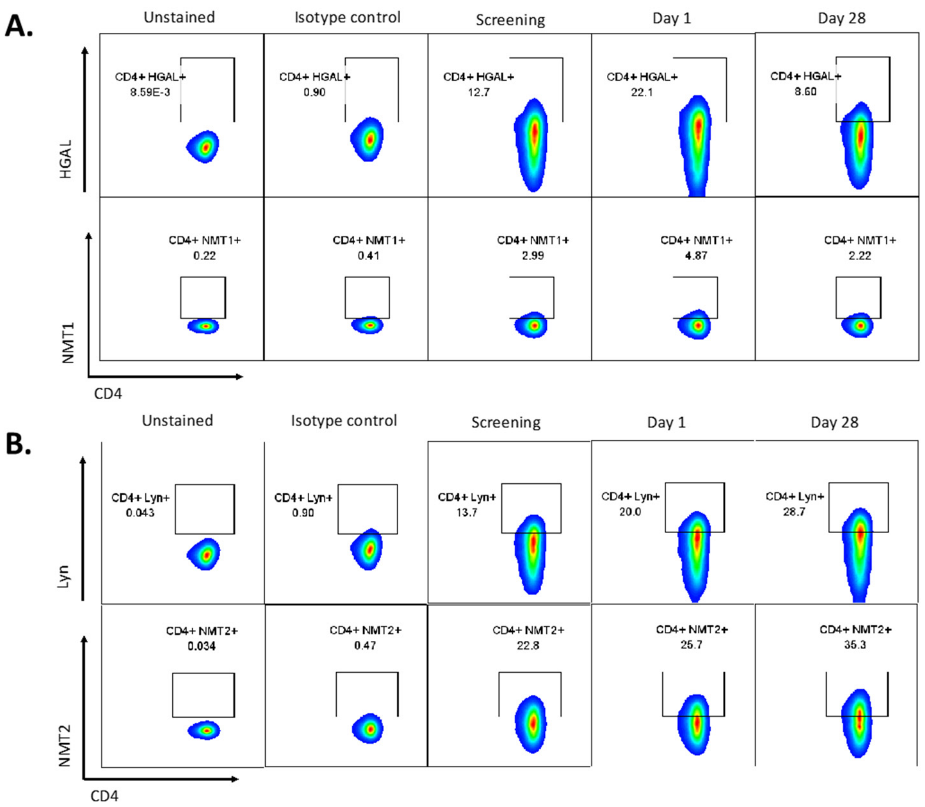

Figure S2. Impact of PCLX-001 treatment on intracellular levels of NMT1, NMT2, HGAL and Lyn in the CD4+ T cells. PBMC were isolated from patient 1 during screening, Day 1 and after 28 days of treatment with PCLX-001 and analysed by flow cytometry. Intracellular staining allowed us to analysed HGAL, NMT1 (**A**), Lyn and NMT2 levels in CD4+ T cell population (**B**).

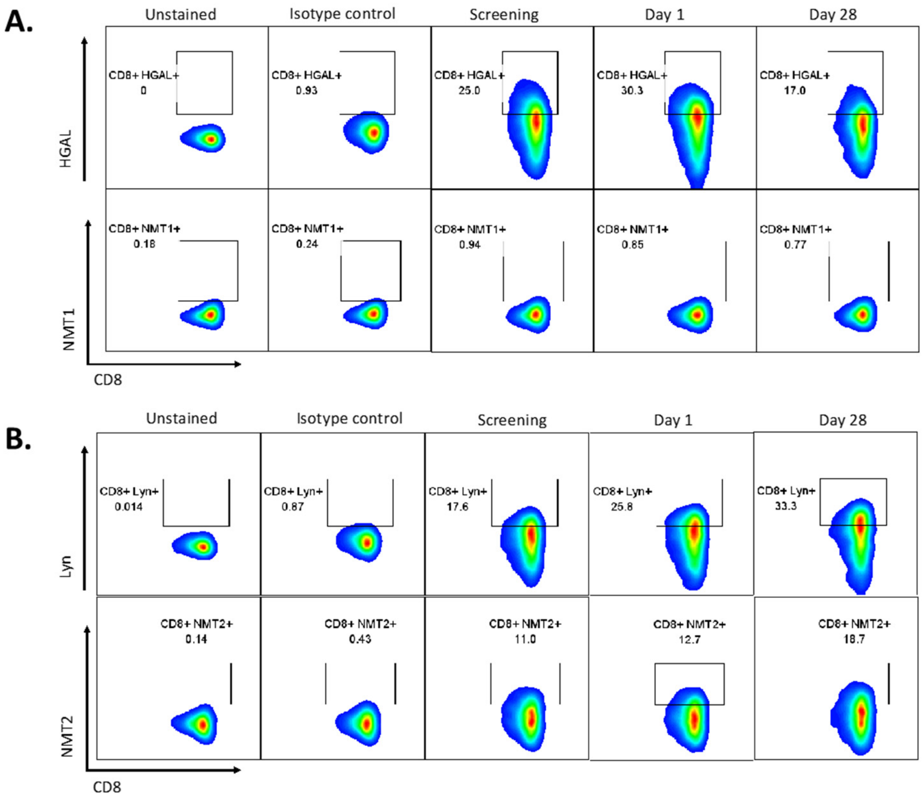

Figure S3. Impact of PCLX-001 treatment on intracellular levels of NMT1, NMT2, HGAL and Lyn in CD8+ T cells. PBMC were isolated from patient 1 during screening, Day 1 and after 28 days of treatment with PCLX-001 and analysed by flow cytometry. Intracellular staining allowed us to analysed HGAL, NMT1 (**A**), Lyn and NMT2 levels in CD8+ T cell population (**B**).

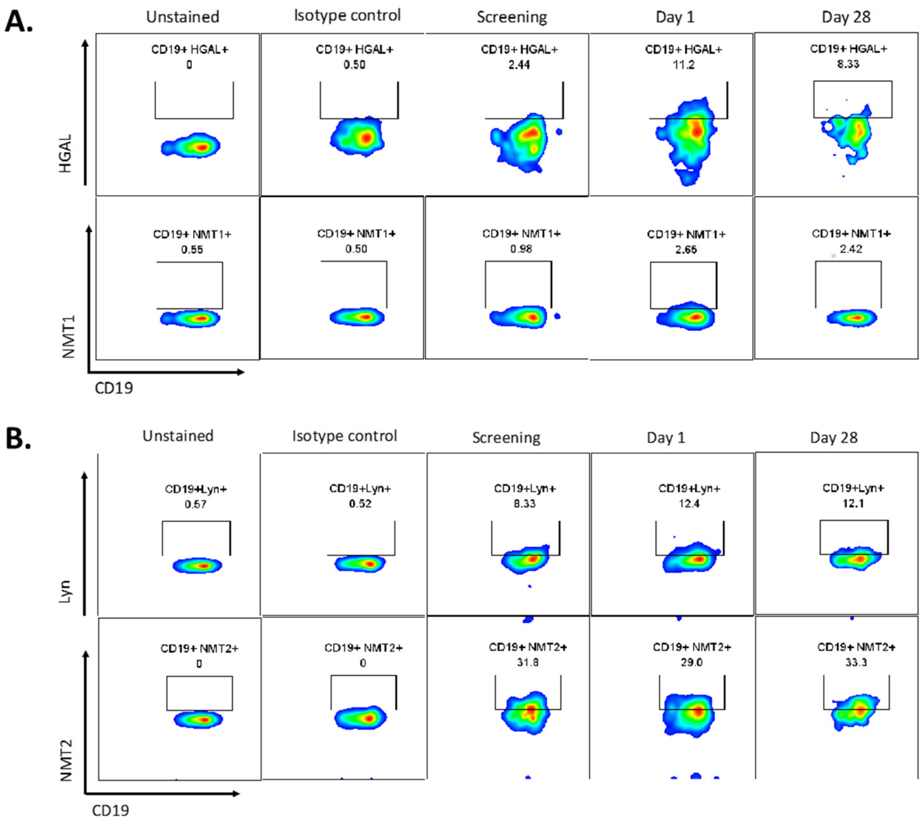

Figure S4. Impact of PCLX-001 treatment on intracellular levels of NMT1, NMT2, HGAL and Lyn in CD19+ B cells. PBMC were isolated from patient 1 during screening, Day 1 and after 28 days of treatment with PCLX-001 and analysed by flow cytometry. Intracellular staining allowed us to analysed HGAL, NMT1 (**A**), Lyn and NMT2 levels in CD19+ B cell population (**B**).

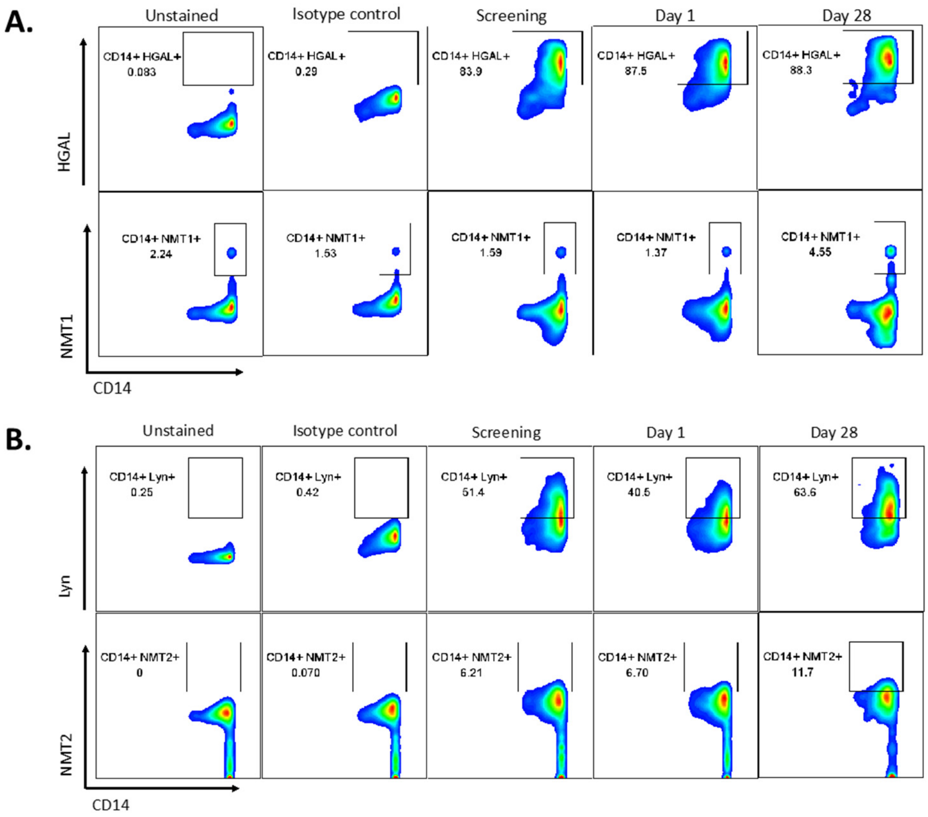

Figure S5. Impact of PCLX-001 treatment on intracellular levels of NMT1, NMT2, HGAL and Lyn in CD14+ monocytes. PBMC were isolated from patient 1 during screening, Day 1 and after 28 days of treatment with PCLX-001 and analysed by flow cytometry. Intracellular staining allowed us to analysed HGAL, NMT1 (**A**), Lyn and NMT2 levels in CD14+ monocyte population (**B**).
